# Cross-Reactive Antibodies to the NS1 Protein of Omsk Hemorrhagic Fever Virus Are Absent in the Sera of Patients with Tick-Borne Encephalitis

**DOI:** 10.3390/v16071032

**Published:** 2024-06-27

**Authors:** Bogdana I. Kravchuk, Yana A. Khlusevich, Galina S. Chicherina, Valeriy V. Yakimenko, Elena I. Krasnova, Nina N. Tikunova, Andrey L. Matveev

**Affiliations:** 1 Institute of Chemical Biology and Fundamental Medicine, Siberian Branch of Russian Academy of Sciences, 630090 Novosibirsk, Russia; khlusevichjana@mail.ru (Y.A.K.); tikunova@niboch.nsc.ru (N.N.T.); 2 Institute of Systematics and Ecology of Animals, Siberian Branch of Russian Academy of Sciences, 630091 Novosibirsk, Russia; chicherinagalina@bk.ru; 3 Omsk Research Institute of Natural Foci Infections, 644080 Omsk, Russia; yakimenko_vv@oniipi.org; 4 Federal State Budgetary Educational Institution, Higher Education “Russian University of Medicine”, The Ministry of Health of the Russian Federation, 630091 Novosibirsk, Russia; krasnova-inf@rambler.ru

**Keywords:** NS1 protein, Omsk hemorrhagic fever virus, OHFV, tick-borne encephalitis virus, TBEV, flaviviruses, flavivirus diagnosis

## Abstract

Omsk hemorrhagic fever virus (OHFV) is a member of the tick-borne encephalitis virus (TBEV) complex of the *Flaviviridae* family. Currently, there are no data on the cross-reactivity of antibodies to the NS1 proteins of OHFV and TBEV. Such data are of major interest for monitoring viral encephalitis of unknown etiology due to the increasing geographical distribution of OHFV. In this study, a recombinant OHFV NS1 protein was produced using the *Escherichia coli* expression system and purified. The recombinant OHFV NS1 protein was recognized by specific mice immune ascetic fluids to the native OHFV NS1 protein. A Western blot analysis and ELISA of the recombinant NS1 proteins of OHFV and TBEV were used to study the cross-reactivity of antibodies from immune ascites fluid obtained from OHFV-infected mice and mAbs against TBEV NS1. Anti-TBEV NS1 mouse monoclonal antibodies (mAbs) have been shown to not be cross-reactive to the OHFV NS1 protein. Sera from patients with confirmed tick-borne encephalitis (TBE) were examined by ELISA using recombinant OHFV NS1 and TBEV NS1 proteins as antigens. It was shown for the first time that cross-reactive antibodies to the OHFV NS1 protein were not detected in the sera of TBE patients, whereas the sera contained antibodies to the TBEV NS1 protein.

## 1. Introduction

Omsk hemorrhagic fever (OHF) is a zoonotic infectious disease that was registered only in several regions of Russian Siberia until 2022 [1]. The infection is caused by the Omsk hemorrhagic fever virus (OHFV, *Orthoflavivirus omskense*), which contains a single-stranded (+) RNA genome [2]. OHFV belongs to the flavivirus complex of the tick-borne encephalitis virus (TBEV) of the *Flaviviridae* family. During the incubation period, infected individuals develop nonspecific flu-like symptoms, followed by the appearance of primary specific symptoms, including headache, cough, nausea, chills and muscle pain, as well as gastrointestinal symptoms, subconjunctival bleeding, nasal, gingival and uterine bleeding [1]. Skin hemorrhages then appear, accompanied by fever (39 °C–40 °C). In addition, the disease leads to skin hyperesthesia and a rash on the upper part of the body. In 30–50% of patients, encephalitic symptoms and meningeal triad are manifested. The diagnosis of this infection is symptomatic, and in the absence of hemorrhagic manifestations, this pathology remains undiagnosed [1]. OHFV is transmitted to humans by bites of ticks from the *Dermacentor* genus (*D. reticulatus* and *D. marginatus*) and *Ixodes* genus (*I. persulcatus*), by direct contact with infected animals and by airborne or foodborne transmission [3].

In 2022, data on the wide spread of OHFV across the territory of the Republic of Kazakhstan appeared [4]. The virus was found in three regions, including Alma-Ata and West Kazakhstan Oblast, which do not border the regions endemic for OHFV in Russian Siberia and are 1000 km or more away from them [2]. For this reason, the spread of OHFV in new areas endemic for TBEV may lead to complications in the serologic surveillance of seasonal TBEV activity and diagnosis of patients infected with TBEV. In this regard, it is required to develop test systems for the diagnosis of OHF, including a retrospective epidemiological analysis of patients with clinical signs of spring–summer tick-borne meningoencephalitis, in whom TBEV or antibodies to it were not detected.

For the detection of OHFV, as for other flaviviruses, the use of RT-PCR-based methods are limited due to the peculiarities of the OHFV life cycle in the human body [1,5]. In this regard, the diagnosis of OHF is currently based on clinical features and the presence of cross-reactivity between antibodies against glycoproteins E of TBEV, and OHFV excludes the use of existing serologic kits and neutralization assay for the differential diagnosis of these infections [6,7]. An additional difficulty for the differential diagnosis of OHF and TBE is the possibility of hemorrhagic forms of TBE, which have been described previously [8].

It is known that the most suitable target for the differential serologic diagnosis of flavivirus infections is the non-structural flavivirus protein NS1 [6,9,10]. NS1 is a highly conserved protein with a molecular mass of approximately 46 kDa, which is detected in the serum of flavivirus-infected persons [6,9,11]. Initially, the NS1 protein is synthesized intracellularly in a monomeric form; then, it forms a dimeric form upon maturation in the endoplasmic reticulum and is transported to the surface of infected cells [12]. The hexameric form of the NS1 protein of flaviviruses is secreted and circulates in the bloodstream of infected individuals [12]. The intracellular form of NS1 plays a role in viral genome replication and virion maturation, whereas the extracellular hexameric form is capable of triggering various immunologic responses, including the formation of anti-NS1 antibodies [13]. In recent years, the NS1 protein has been used as an antigen for the early diagnosis of mosquito-borne flavivirus infections by ELISA, as it is detected in infected individuals on days 6–8 [6,9,10]. It is known that the NS1 protein appears in the serum of infected persons before the immune response to protein E [14]. In addition, test systems based on the NS1 antigen are currently used for epidemiologic diagnosis, including the differential diagnosis of the Saint Louis encephalitis and West Nile viruses [15]. 

The possibility of using the NS1 protein as an ELISA antigen for the differential diagnosis of tick-borne flavivirus infections has not been previously investigated. In addition, the cross-reactivity of antibodies against the NS1 protein of TBEV and sera from TBE-infected patients to the NS1 protein of OHFV has not been studied. In this study, the recombinant OHFV NS1 protein was produced in *Escherichia coli* and purified. Monoclonal antibodies (mAbs) against TBEV NS1 did not bind the recombinant OHFV NS1 protein, whereas mAbs against the native OHFV NS1 recognized it. In addition, cross-reactive antibodies against OHFV NS1 were not found in the sera of patients with confirmed TBE.

## 2. Materials and Methods

### 2.1. Sera from Patients

Sera from patients (54% males and 46% females) hospitalized in Novosibirsk Infectious Diseases Clinical Hospital No. 1 between April and September 2017–2019 with TBE and sera from healthy volunteers were used in this study. These serum samples have been previously characterized for the presence of anti-TBEV antibodies [16]. Patient selection criteria were as follows: (i) a history of tick bites; (ii) the presence of symptoms of infectious diseases; (iii) patient age more than 18 years. All patients underwent appropriate medical examinations, including the serological detection of TBE. Volunteers were young healthy adults without chronic diseases (including autoimmune diseases) who had not been infected and/or hospitalized for at least six months. All patients and volunteers participating in this study gave informed consent. This study was approved by the ethical committee of the Novosibirsk Infectious Diseases Clinical Hospital No. 1. Sera were stored at −70 °C. These serum samples have been previously characterized for the presence of anti-TBEV protein E antibodies by ELISA and for TBEV RNA by RT-PCR [16]. 

### 2.2. Phylogenetic Analyses of OHFV NS1 Gene and In Silico Structural Analysis of NS1 Protein of OHFV Strain 186–1964

The OHFV NS1 gene was amplified by a reverse transcription reaction using the OT-M-MuLV-RH reagent kit (Biolabmix, Novosibirsk, Russia) with random hexaprimers; total RNA was isolated from the inactivated brain lysate of a BALB/c mouse infected with OHFV; strain 186–1964 was used as a template. PCR fragments encoding the NS1 protein of OHFV (1–352 aa) was obtained using primers Start_NS1_OHFV_59_pET_U26 5′-CCGTTGGATCCGACGTTGGATGTGCTGTGTGGACACTGA-3′ and End_NS1_OHFV_58_pET_L21 5′-GGCTTTGAATTCCCAGCCACCACCACCATCGAGCGCGCAC-3′ and cDNA as a matrix. For phylogenetic analyses, the sequence of the NS1 gene of OHFV, strain 186–1964, was determined and aligned with those of other OHFV strains and several TBEV strains including the European, Far-Eastern and Siberian subtypes via the MUSCLE algorithm in MEGA X [17]. The maximum likelihood phylogenetic tree was constructed using the TN93+G+I substitution model with 1000 bootstrap iterations in MEGA X.

The putative three-dimensional (3D) structure of the OHFV NS1 protein was predicted using the AlphaFold2 algorithm [18] (https://colab.research.google.com/github/sokrypton/ColabFold/blob/main/AlphaFold2.ipynb, accessed on 11 September 2023). The UCSF Chimera molecular visualizer, version 1.15, was used to visualize ribbon and surface representations of the OHFV NS1 protein [19].

### 2.3. The Construction of the Plasmid Encoding the OHFV NS1 Protein

Bacterial strains *E. coli* XL1-Blue and *E. coli* BL21 (DE3) that were used to construct and produce the target proteins were obtained from the Collection of Extremophile Microorganisms and Type Cultures (CEMTC) of ICBFM SB RAS.

The obtained PCR fragment containing the OHFV NS1 gene and the expression plasmid pET-32a(+) were cleaved by the restriction endonucleases *Bam*HI and *Eco*RI (Sibenzyme, Novosibirsk, Russia) and combined in a ligation reaction. *E. coli* XL1-Blue cells (recA1, endA1, gyrA96, thi, hsdR17 (rK−, mK+), supE44, relA1, lac, [F′, proAB+, laclqZΔM15, Tn10 (Tetr)]) were transformed with the resulting ligation products and seeded on LB agar with ampicillin at a dose of 50 μg/mL and cultured. Individual colonies of *E. coli* cells containing plasmid pET-32a(+)_NS1OHFV were tested by PCR using the same primers. PCR amplification conditions were as follows: 5 min at 95 °C, followed by 30 cycles of 30 s at 95 °C, 20 s at 56 °C, 1.5 min at 72 °C and a final elongation of 6 min at 72 °C. The obtained PCR products were assessed by electrophoresis in 1% agarose gel. The accuracy of the insertion of the gene encoding the OHFV NS1 protein was confirmed by Sanger sequencing using primers pETseq-dir 5′-TGCTAGTTAGTAGTATTGCTCAGCG-3′ and pETseq-rev 5′-GGTTCTGGTTCTGGTTCTGGTTCTGGCCATA-3′. The resulting plasmid was pET-32a(+)_NS1OHFV, which encodes the OHFV NS1 protein with a His-tag at the C-terminus.

In the recombinant expression plasmid pET-32a(+)_NS1OHFV, the NS1 gene is located directly after the Trx coding sequence in the same open reading frame. Meanwhile, the sequence encoding the 6His-tag, which is required for purification using metal chelate chromatography, is located directly after the gene. Thus, the expressed recombinant NS1 protein contains a Trx tag at the N-terminus, a 6His tag at the C-terminus and a thrombin cleavage site between Trx and NS1 sequences.

To produce the OHFV NS1 protein in eukaryotic cells, the gene of this protein was inserted into the expression plasmid pOptiCAG as described earlier [20]. HEK 293 cells were transfected by the resulting plasmids pOptiCAG-NS1 and pOptiCAG-RBD using PEIpro (Polyplus, Illkirch, France) according to the manufacturer’s instructions. The recombinant OHFV NS1 protein was purified from culture media using Ni-NTA resin (Qiagen, Hinden, Germany)

### 2.4. The Optimization of the OHFV NS1 Expression

*E. coli* strain BL21 (DE3) cells (F-, ompT, hsdSB (rB−, mB−), dcm, gal, λ(DE3), pLysS, Cmr) were transformed with the plasmid pET-32a(+)_NS1OHFV, cultured in LB medium in the presence of ampicillin at a concentration of 50 μg/mL. When the cell culture reached OD_600_ = 0.5, isopropyl β-D-1-thiogalactropyranoside (IPTG) at a concentration of 100 μM was added. After induction, cells were grown at 30 °C overnight. The production of the recombinant OHFV NS1 protein in cell lysates was assessed using 12.5% PAGE. 

To select the optimal conditions for OHFV NS1 protein production, different IPTG concentrations (1 μM, 10 μM, 100 μM and 1000 μM) were used to induce the transcription of the target gene when the cell culture reached OD_600_ = 0.5, and the cells were cultivated at different temperatures varying from 12 °C to 30 °C. At 4 h and 11 h after induction, cells were centrifuged at 3000× *g* for 10 min. The cell pellet was resuspended in 50 mM Tris-HCl, pH 8.0, and disrupted using the ultrasonic homogenizer Sonopuls hd 2070 (Bandelin, Berlin, Germany) for 2 min at 30% amplitude, with 10 s of sonication and 10 s of a rest period. The obtained suspension was centrifuged at 16,000× *g* for 10 min, after which the supernatant containing soluble cytoplasmic proteins was transferred to a new tube. The precipitate containing the inclusion body fraction was dissolved in 50 mM Tris-HCl, pH 8.0. The resulting cell fractions were analyzed by 12.5% PAGE under reducing conditions. 

### 2.5. The Purification of the Recombinant OHFV NS1 Protein

*E. coli* BL21 (DE3)/pET-32a(+)_NS1OHFV cells were cultivated under the previously selected conditions and centrifuged at 9000× *g* for 30 min. The precipitate was dissolved in a wash buffer containing 1 mM phenylmethylsulfonyl fluoride (PMSF), 2 M urea, 20 mM Tris-HCl and 500 mM NaCl, sonicated for 15 min as described previously and centrifuged at 15,000× *g* for 10 min. Next, the precipitate was dissolved in the inclusion body solubilizing buffer (pH 7.4, 20 mM Tris-HCl, 6 M urea, 1 mM β-ME, 5 mM imidazole, 500 mM NaCl), sonicated as described above and centrifuged at 12,000× *g* for 15 min. The recombinant OHFV NS1 protein was purified from inclusion bodies using Ni-NTA Sepharose (Qiagen, Venlo, The Netherlands), according to the manufacturer’s protocol. The column containing Ni-NTA agarose was equilibrated with a buffer B (3 M urea, 500 mM NaCl, 20 mM Tris-HCl, 1 mM β-ME, pH 8.0). The supernatant containing solubilized inclusion bodies was applied to the column and washed with the buffer B. The recombinant OHFV NS1 protein was eluted with buffer B, containing 500 mM imidazole, and concentrated using an Amicon Ultra-4 centrifuge filter unit (Millipore, Burlington, MA, USA) with a cutoff threshold of 10 kDa. The recombinant OHFV NS1 protein was consistently dialyzed against three refolding buffers (300 mM NaCl, 200 mM sucrose, 1 mM reduced glutathione (GSH), 0.2 mM oxidized glutathione (GSSG) and 0.1% Triton X-100, pH 7.5) containing consistently decreasing concentrations of urea (2 M, 0.5 M and 0 M urea, respectively) and imidazole (250 mM, 100 mM and 20 mM, respectively). Finally, the purified recombinant OHFV NS1 protein was dialyzed in a storage buffer (50 mM Tris-HCl, pH 8.0, containing 10 mM imidazole, 300 mM NaCl and 200 mM sucrose). The purified recombinant OHFV NS1 protein was analyzed using 12.5% PAGE. Protein purity was determined using the Gel Doc XR+ gel documentation system (Bio-Rad, Hercules, CA, USA) with ImageLab 3.0 software (Bio-Rad, Hercules, CA, USA). Protein concentration was evaluated using the Qubit protein assay kit (Thermo Fisher Scientific, Waltham, MA, USA) on a Qubit 4 fluorometer (Thermo Fisher Scientific, Waltham, MA, USA). The obtained proteins were stored at a concentration of at least 0.5 mg/mL.

### 2.6. Western Blot Analysis

The lysates of *E. coli* cells producing the OHFV NS1 protein were separated using 12.5% PAGE and then transferred to a nitrocellulose membrane (Bio-Rad, Hercules, CA, USA). After blocking the nonspecific binding sites with 3% bovine serum albumin solution (BSA, Amresco, Solon, OH, USA), the membrane was incubated with sera obtained from TBE patients at a dilution of 1:800 (N = 26) or with 0.02 mg anti-TBEV FVN-NS1-3, FVN-NS1-6, FVN-NS1-44, FVN-NS1-290 and FVN-NS1-299 (N = 5). These mAbs have been previously obtained by a known hybridoma technology using mice infected with TBEV strain Sofjin (Far-Eastern subtype) [21]. The membrane was then incubated with Anti-Mouse IgG (Fc-specific)–Peroxidase conjugated polyclonal antibodies produced in rabbits (Biosan, Novosibirsk, Russia). Immune complexes were revealed using 4-chloro-1-naphthol (Applichem, Darmstadt, Germany). Immune ascitic pooled fluids obtained from OHFV-infected mice were used as a positive control.

### 2.7. ELISA

For indirect ELISA, 1 μg/mL of anti-His tag mAb.H1 (Biolabmix, Novosibirsk, Russia) was sorbed into the wells of 96-well polystyrene plates (Greiner, Kremsmünster, Austria); then, after blocking the nonspecific binding sites with 5% skim milk solution, 1 μg of the OHFV NS1 protein or TBEV NS1 protein (as a positive control) was added. After incubation, sera obtained from TBE-positive patients at a dilution of 1:500 (N = 26) or individual mAb against the TBEV NS1 protein (N = 5) at a concentration of 10 μg/mL were added. After washing, wells were incubated with Anti-Mouse IgG (Fc-specific) HRP conjugated polyclonal antibodies produced in rabbits (Biosan, Novosibirsk, Russia) or Anti-Human IgG (Fc specific) HRP conjugated mAb X-53 (Biosan, Novosibirsk, Russia) for one hour at 37 °C. Immune complexes were revealed using tetramethylbenzidine-3,3,5,5 (TMB, Applichem, Solon, OH, Germany). Absorbance was measured at a wavelength of 450 nm using a microplate reader (Bio-Rad, Hercules, CA, USA).

To determine the cutoff level for ELISA with sera from TBE patients, sera obtained from conditionally healthy donors who had not previously had TBE were used. The mean optical density level of the control sera plus one standard deviation was taken as the cutoff. The ELISA signal levels of sera from TBE patients above the cutoff were considered positive. 

For competitive ELISA, immune ascitic pooled fluids obtained from OHFV-infected mice were pre-incubated with 5 µg of purified OHFV NS1 obtained using *E. coli* cells in 1:000 dilution for 1 h at 37 °C. The purified OHFV NS1 protein obtained using HEK 293 cells was used as antigen.

### 2.8. Statistics

Statistical analysis was carried out using a one-way ANOVA using the Statistica 10 software package (StatSoft Inc., Tulsa, OK, USA).

## 3. Results

### 3.1. The Phylogenetic Analysis and In Silico Modeling of the OHFV NS1 Protein

**Figure 1:**
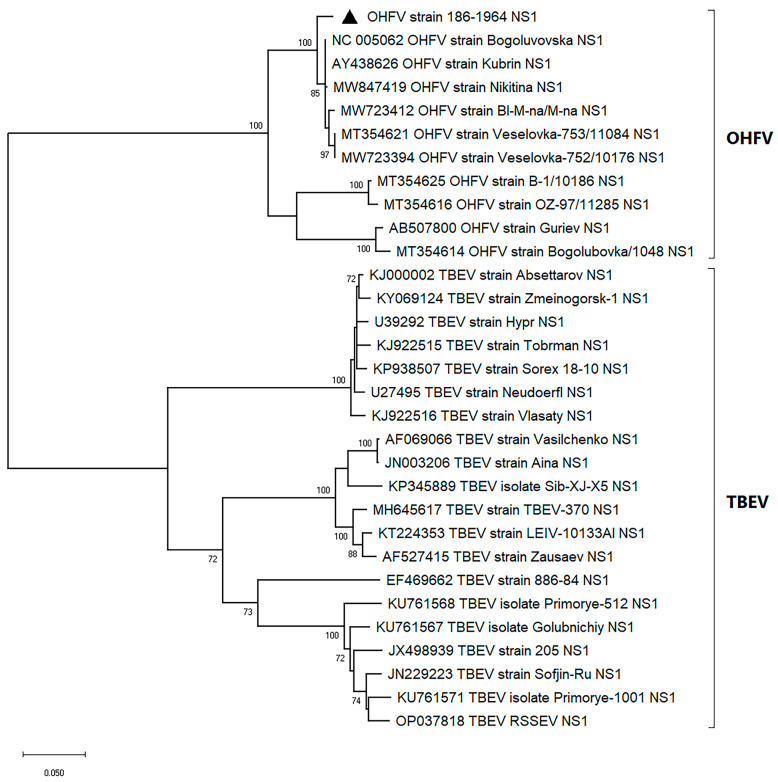
The phylogram constructed by the ML method based on nucleotide sequences encoding OHFV NS1 and TBEV NS1 proteins, TN93+G+I substitution model with 1000 bootstrap iterations in MEGA X. Significant bootstrap values (>70%) are shown on the nodes. The sequence determined in this study is highlighted by a triangle.

**Figure 2:**
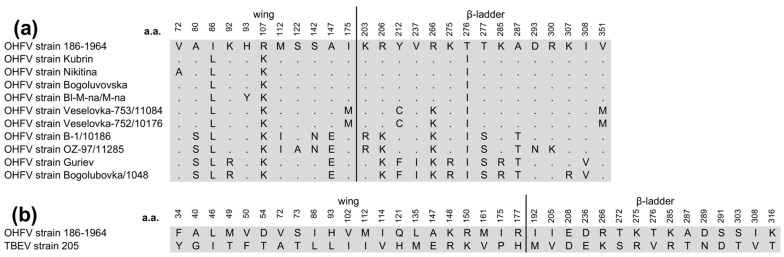
Condensed alignment based on the aa sequence of the NS1 protein OHFV, strain 186–1964, obtained in this study, and NS1 proteins of 10 strains of OHFV (**a**) and protein TBEV NS1 strain 205 (**b**).

The sequencing of the gene encoding the OHFV NS1 protein, strain 186–1964, and the subsequent phylogenetic analysis showed that the sequence of this gene clustered with the NS1 gene sequences of previously described OHFV strains (Figure 1). The nucleotide identity (NI) of the investigated NS1 gene with the closest published strains of OHFV Kubrin and OHFV Bogoluvovska was 98.2% (19 nucleotide substitutions). This resulted in only three amino acid (aa) substitutions, two of which did not change the class of the aa residue (Figure 2a). Expectedly, the sequence similarity of the NS1 gene sequence of OHFV 186–1964 to the corresponding TBEV genes was lower, and the NI value was 80.1% (210 nucleotide substitutions, Figure 2b).

It is known that the flaviviral NS1 protein contains three domains: the β-roll domain, 1–29 aa; the wing domain, 30–180 aa; and the β-ladder domain, 181–352 aa [22]. It was shown that “β-roll” participates in NS1 dimerization and is hidden inside the NS1 dimer, while aa residues from other domains are exposed on the surface of the NS1 dimer. Notably, all aa residues, which are different between OHFV NS1 and TBEV NS1, are located predominantly in the wing and β-ladder domains (21 and 15 aa, respectively) exposed on the surface of the NS1 dimer (Figure 2b). To predict the glycosylation site on the OHFV NS1 protein, the NetNglyc server predicts N-Glycosylation sites in human protein [23]. It was shown that the OHFV NS1 protein contains two sites of N-glycosylation (85-N and 207-N), which correspond to the predicted glycosylation sites of the TBEV NS1 protein. Thus, obtaining a deglycosylated OHFV NS1 protein may reduce cross-reactivity to TBEV NS1 protein antibodies.

**Figure 3:**
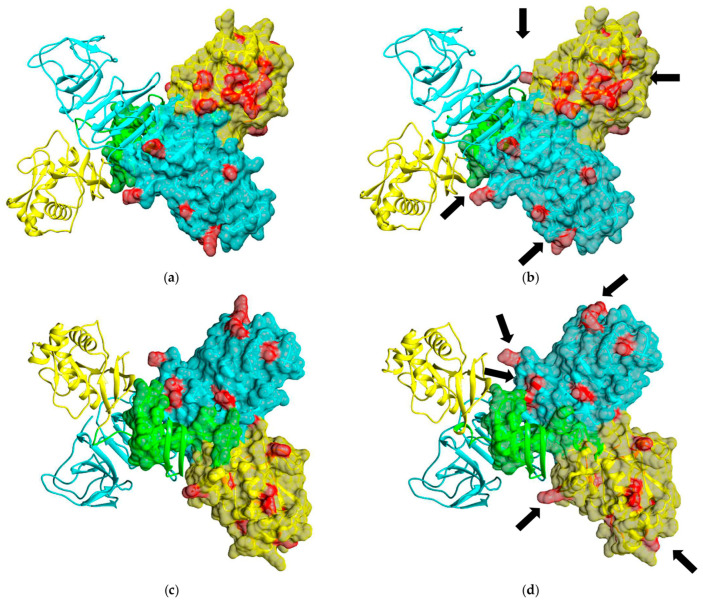
The predicted 3D structures of the OHFV NS1 and TBEV NS1 protein dimers obtained using the AlphaFold2 algorithm. Domains 1, 2 and 3 are marked in green, yellow and blue, respectively. Aa residues that differ between OHFV NS1 and TBEV NS1 proteins are highlighted in red. (**a**) Top view of the OHFV NS1 protein dimer; (**b**) top view of the TBEV NS1 protein dimer; (**c**) bottom view of the OHFV NS1 protein dimer; (**d**) bottom view of the TBEV NS1 protein dimer. The molecular coordinates of the putative OHFV NS1 protein were displayed using the UCSF Chimera, version 1.15.

To analyze the arrangement of aa substitutions on the surface of NS1 OHFV, the 3D structures of the OHFV NS1 and TBEV NS1 proteins were predicted using the AlfaFold2 algorithm [18]. The modeling results (Figure 3) indicated that the OHFV NS1 protein has a structure similar to the resolved structures of the NS1 proteins of Zika virus, West Nile virus, Japanese encephalitis virus and dengue virus, for which the 3D structures were previously obtained [24,25,26]. The NS1 proteins of OHFV form a homodimer, each consisting of three domains. As shown above, all differences in aa residues are in domains 2 and 3, which are marked in red in Figure 3. Almost all of the differing aa are located on the surface of the OHFV NS1 protein and may form different epitopes for antibodies than TBEV NS1. Thus, this analysis indicates that the OHFV NS1 protein could potentially be used for the differential diagnosis of OHF and TBE.

NS1 is an important flavivirus protein that plays a central role in efficient viral RNA replication. This protein accumulates intracellularly and is secreted from infected cells. The soluble hexamer of the flaviviral NS1 protein circulates in serum and other body fluids and therefore can be detected. Since antibodies against this protein are produced during, they can be used for serologic diagnostics. 

### 3.2. The Production of the OHFV NS1 Protein in E. coli Cells

In the recombinant expression plasmid pET-32a(+)_NS1OHFV, the NS1 gene (GenBank accession number PP525060) is located directly after the Trx coding sequence in the same open reading frame. Meanwhile, the sequence encoding the 6His-tag, which is required for purification using metal chelate chromatography, is located directly after the gene. Thus, the expressed recombinant NS1 protein contains a Trx tag at the N-terminus, a 6His tag at the C-terminus and a thrombin cleavage site between Trx and NS1 sequences.

**Figure 4:**
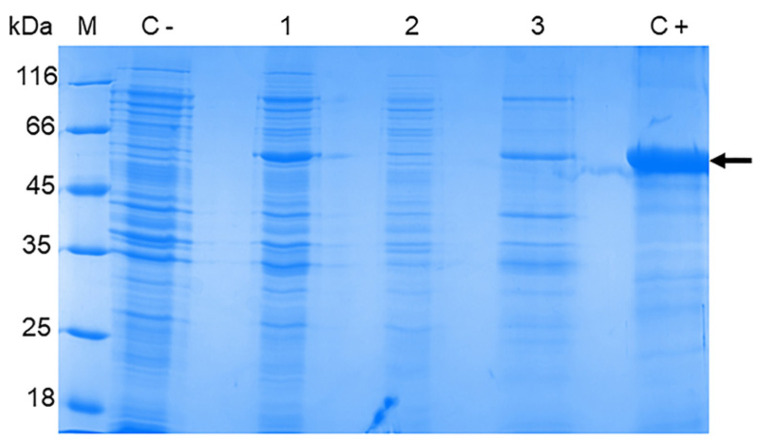
SDS-PAGE of lysates of *E. coli* BL21 (DE3) cells transformed with the plasmid pET-32a(+)_NS1OHFV (1), cytoplasmic fraction (2) and inclusion body fraction (3). The BL21 (DE3) cells transformed with pET-32a(+) plasmid were used as control (1). Purified TBEV NS1 protein [21] was applied as a positive control (5). M—molecular mass marker of proteins.

To obtain the recombinant OHFV NS1 protein, *E. coli* BL21 (DE3) cells were transformed with the obtained plasmid pET-32a(+)_NS1OHFV and induced. The lysates of induced BL21 (DE3)/pET-32a(+)_NS1OHFV cells were fractionated using 12.5% SDS-PAGE. The electrophoretic mobility of the obtained recombinant OHFV NS1 corresponded to the theoretically predicted ~ 58 kDa and also corresponded to the electrophoretic mobility of recombinant TBEV NS1 obtained previously using a similar plasmid vector [20]. The OHFV NS1 protein was found to be located in the inclusion body fraction (Figure 4). 

**Figure 5:**
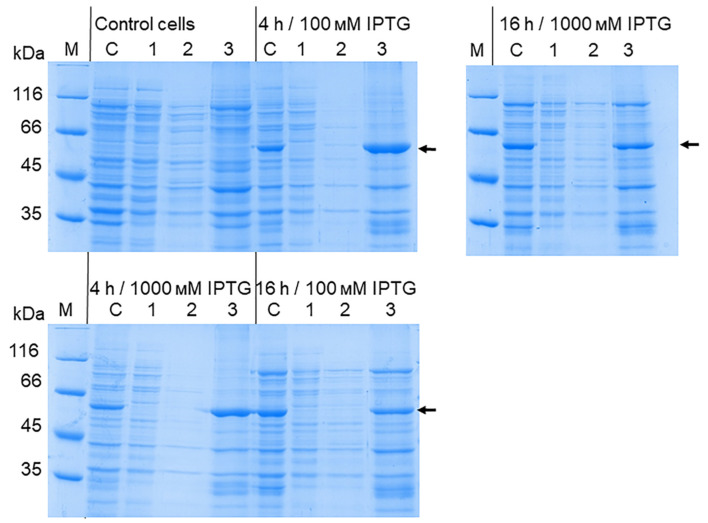
SDS-PAGE of lysates of *E. coli* BL21 (DE3)/pET-32a(+)-NS1OHFV cells producing Trx-OHFV NS1 protein after optimization of induction conditions using different concentrations of IPTG; cultured for 1, 2, 4 and 16 h at 18 °C and 30 °C. 1—cells induced with 0.001 mM IPTG; 2—cells induced with 0.01 mM IPTG; 3—precipitate of cells induced with 0.1 mM IPTG; 4—lysate of cells induced with 1 mM IPTG. M—PageRuler™ Unstained Protein Ladder (#26613, Thermo Fisher scientific, Waltham, MA, USA).

The production of the recombinant OHFV NS1 protein in either *E. coli* cells or eukaryotic expression systems has not been previously described. To increase the solubility of the recombinant protein, we optimized the cultivation conditions similar to those previously used to obtain the soluble TBEV NS1 protein strain Sofjin in *E. coli* [21]. For this purpose, *E. coli* BL21 (DE3) cells transformed with the pET-32a(+)-NS1OHFV plasmid were grown at various IPTG concentrations, temperature and duration of cultivation after induction. The results showed that the induction of the transformed cell culture with 0.01 mM IPTG for 11 h at 12 °C resulted in the maximum yield of the soluble OHFV NS1 protein. The induction of the transformed culture with 0.1 mM IPTG for 11 h at 30 °C resulted in the maximum yield of the OHFV NS1 protein; however, almost all of the NS1 protein was localized in the inclusion body fraction (Figure 5). The yield of the soluble OHFV NS1 protein was low, so the refolded protein from the inclusion body fraction was obtained for further studies.

### 3.3. OHFV NS1 Protein Isolation and Purification 

**Figure 6:**
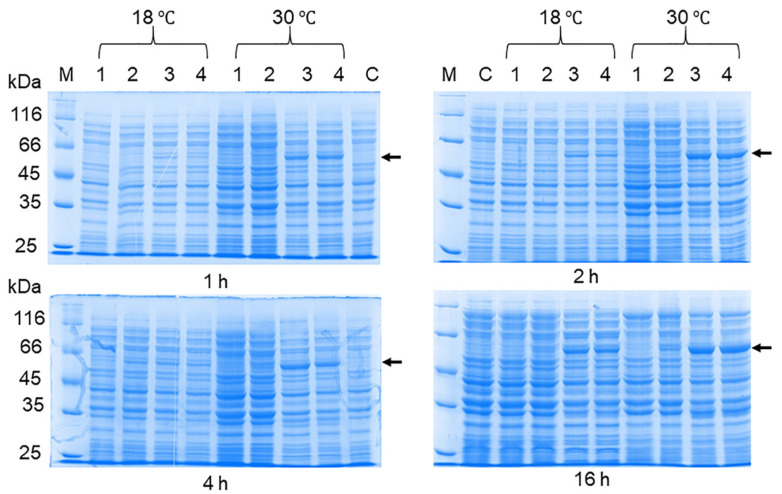
SDS-PAGE of *E. coli* BL21 (DE3)/pET-32a(+)-NS1OHFV cell lysates cultured under different conditions and their different fractions. M—protein molecular weight marker, 1—initial cell lysates, 2—soluble cytoplasm, 3—fraction of inclusion bodies after refolding in 2 M urea, 4—fraction of inclusion bodies after refolding in 6 M urea.

Protein purification was performed using Ni-NTA agarose from the inclusion body fraction, similar to the previously described protocol for the TBEV NS1 [14]. Buffers with increasing concentrations of urea were used to isolate the OHFV NS1 protein from inclusion bodies (Figure 6). The maximum dissolved protein content was observed in a wash buffer containing 6 M urea (Figure 6). The NS1 protein was purified by metal chelate chromatography on Ni-NTA agarose under denaturing conditions; bound protein was eluted with buffer A containing 500 mM imidazole (Figure 6). The OHFV NS1 protein was dialyzed with a refolding buffer containing reduced (GSH) and oxidized (GSSG) glutathione and sucrose to promote the acquisition of the correct conformation of OHFV NS1. The productivity was 80 mg of the purified OHFV NS1 protein per 1 L of cell culture. The purity of the resulting purified OHFV NS1 protein, assessed after PAGE, was about 90%. The electrophoretic mobility of the purified recombinant OHFV NS1 protein was ~58 kDa.

**Figure 7:**
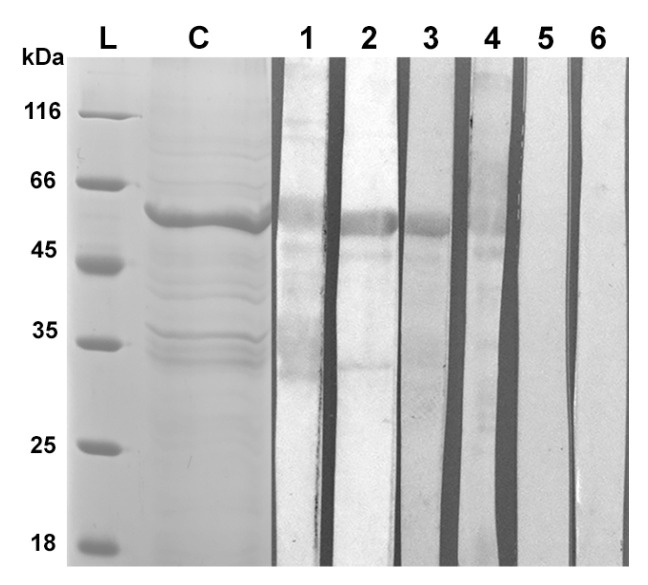
Western blot analysis of the recombinant OHFV NS1 (lines 1–3) and TBEV NS1 (lines 4–6) proteins bound by mAb.His1 (lines 1,4) against the C-terminal His-tag and immune ascites fluids from mice infected with OHFV P-15-2213 (lines 2,5) and OHFV Oz-31_Kd_10866 (lines 3,6).

It has been shown that mAb.His1 against the C-terminal His-tag (Biosan, Novosibirsk, Russia) reveals in the lysate of *E. coli* BL21 (DE3)/pET-32a(+)-NS1OHFV cells a protein with a molecular mass of about 58 kDa, which corresponds to the recombinant OHFV NS1 protein (Figure 7). The TBEV NS1 protein, also possessing a C-terminal His-tag [14], which was previously shown to be detected by the mAb.His1, was used as an antigen control.

### 3.4. The Evaluation of the Antigenic Properties of the Recombinant OHFV NS1 Protein 

**Figure 8:**
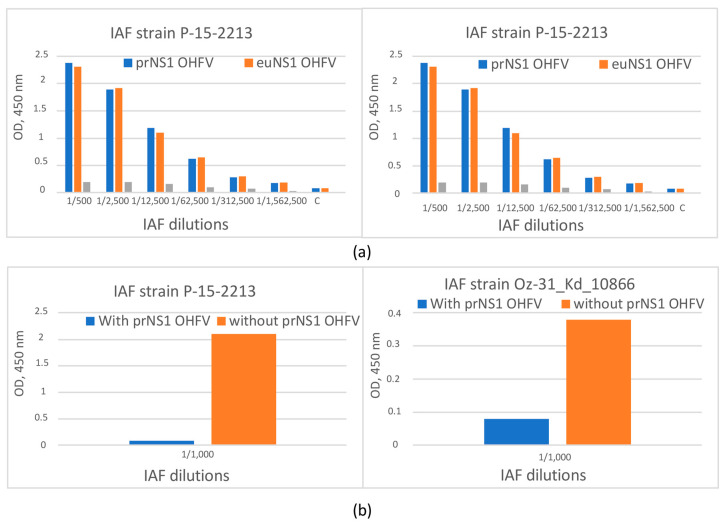
(**a**) ELISA of serial dilution of immune ascetic fluids from mice infected with OHFV strains P-15-2213 and Oz-31_Kd_10866 to bind recombinant OHFV NS1 and TBEV NS1 proteins. (**b**) Competitive ELISA of serial dilution of immune ascetic fluids from mice infected with OHFV strains P-15-2213 and Oz-31_Kd_10866 to bind recombinant OHFV euNS1 protein after incubation with recombinant OHFV prNS1 proteins. prNS1 OHFV—protein, obtained using *E.coli* cells. euNS1 OHFV—protein, obtained using 293 cells.

The antigenic properties of the obtained OHFV NS1 protein were evaluated using ELISA. For this purpose, immune ascites fluids (anti-OHFV IAF) were used that were obtained from mice independently infected with two different OHFV strains (OHFV P-15-2213 and OHFV Oz-31_Kd_10866) [4,27]. The NS1 protein of TBEV strain Sofjin was used as a negative control antigen. Serial dilutions of two anti-OHFV IAFs were tested for their binding to both the recombinant OHFV NS1 (eukaryotic and prokaryotic) and TBEV NS1 proteins using ELISA. Both anti-OHFV IAFs bound recombinant OHFV NS1 proteins at a dilution of at least 1:100,000 (1:4,500,000 for the strain P-15-2213), whereas they revealed recombinant TBEV NS1 strain Sofjin protein at a dilution of 1:500 (Figure 8). In competitive ELISA using NS1 OHFV from 293 cells as antigens, it was demonstrated that the pre-incubation of both anti-OHFV IAFs with the prokaryotic OHFV NS1 protein led to the inhibition of binding these anti-OHFV IAFs to the eukaryotic OHFV NS1 protein. Thus, it was demonstrated that the obtained recombinant OHFV NS1 strain 186–1964 protein retained its antigenic properties after refolding. 

Western blot analysis indicated that both anti-OHFV IAFs and the anti-His-tag mAb.His1 revealed a protein with a molecular mass of approximately 58 kDa, which corresponds to the recombinant OHFV NS1 protein (Figure 7). None of the IAFs, unlike mAb.His1, detected the recombinant TBEV NS1 protein. This is probably due to the small number of similar epitopes on the surface of OHFV NS1 and TBEV NS1 (Figure 7). So, after refolding, the resulting recombinant OHFV NS1 protein retained its conformation epitopes recognizable by specific antibodies against the native OHFV NS1. 

**Figure 9:**
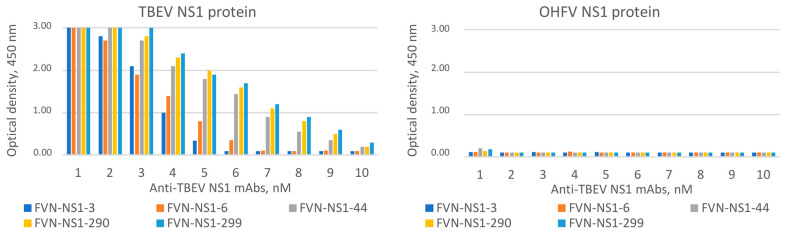
ELISA of serial dilutions of FVN-NS1-44, FVN-NS1-290 and FVN-NS1-299 monoclonal antibodies specific to TBEV NS1 to bind recombinant OHFV NS1 and OHFV NS1 proteins.

The ability of TBEV NS1-specific mAbs FVN-NS1-3, FVN-NS1-6, FVN-NS1-44, FVN-NS1-290 and FVN-NS1-299 to bind the recombinant OHFV NS1 protein was tested. These mAbs have been previously obtained and characterized [21]. In parallel experiments, equal concentrations of OHFV NS1 and TBEV NS1 proteins were detected by serial dilutions of mAb. It was shown that these mAbs recognized only TBEV NS1 and did not bind NS1 OHFV. The obtained data indicate that the epitopes that were bound by mAbs FVN-NS1-3, FVN-NS1-6, FVN-NS1-44, FVN-NS1-290 and FVN-NS1-299 were absent on the OHFV NS1 surface (Figure 9).

Thus, it was confirmed that the purified OHFV NS1 protein retained its antigenic properties, and its antigenic structure is different from that of TBEV NS1, which provides the possibility of using recombinant OHFV NS1 for the differential diagnosis of TBE and OHV infection. 

### 3.5. The Examination of Sera from Patients with TBE for Cross-Reactivity with the OHFV NS1 Protein

**Figure 10:**
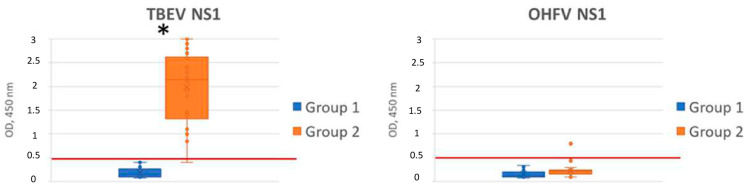
ELISA of human sera for antibodies binding TBEV NS1 and OHFV NS1 proteins. *—ρ < 0.05, (one-way ANOVA). Symbols: blue column—sera from healthy donors without TBE and OHF (group 1), orange column—sera from patients with clinically confirmed TBE (group 2). Red line—cutoff level.

A panel of sera obtained from patients with confirmed TBE was examined for the ability to detect OHFV NS1. At the first step, the cutoff was determined. For this purpose, a panel of sera (N = 96) obtained from healthy volunteers who had no TBE and OHF previously (group 1) was tested. OHFV NS1 and TBEV NS1 were used as antigens. The cutoff value was 0.494. Then, 26 sera obtained from patients with confirmed TBE (group 2) were tested for antibodies to TBEV NS1. Since two serum samples showed a low signal (below cutoff), 24 TBEV NS1-positive sera were selected for further analysis. These positive sera were tested by ELISA for the presence of cross-reactive antibodies to the OHFV NS1 protein. The result indicated that the signal in ELISA was above the cutoff level only in one serum, and in another one, the antibody level was equal to the cutoff, corresponding to a questionable result (Figure 10, Table S1). Thus, the specificity of ELISA using the recombinant OHFV NS1 protein for the differential diagnosis of TBE and OHFV was at least 90%. Thus, more than 95% of TBEV NS1-positive sera were not recognized in ELISA with the recombinant OHFV NS1 protein.

## 4. Discussion

OHFV is a rare, diagnosed virus; the first recorded outbreak was observed in the Siberia region of the USSR between 1945 and 1958, with 972 confirmed cases and more than 1500 cases of suspected OHF. Until 1998, more than 300 additional cases were recorded in the Tyumen, Kurgan, Omsk and Novosibirsk regions of the Russian Federation. After 1998, no new cases of OHF were reported in Russia and other countries, which is probably due to both the lack of specific diagnostics and the probable mild course of the disease [12]. Another likely reason for the absence of new OHF cases is routine TBEV vaccinations. It is known that protective immunity against TBEV can also protect a vaccinated person from OHFV [1]. In 2022, OHF was unexpectedly detected in the new regions of the Republic of Kazakhstan, located more than 1000 km from OHF-endemic areas in Russia. In this regard, it is required to strengthen the epidemiological control of OHFV.

The correct diagnostics of flavivirus infections is difficult, especially in regions where more than one flavivirus is circulating. The cross-reactivity of antibodies to the E glycoprotein of closely related mosquito-borne flaviviruses, such as dengue virus and Zika virus, is known to cause the low specificity of ELISA and serological tests based on the detection of E glycoprotein or antibodies to it [27]. Because of the significant aa differences in the NS1 proteins of these viruses, several ELISA kits have been developed for the detection of antibodies to the NS1 protein of Zika virus, and such test systems showed high specificity [28,29,30,31]. In addition, sera from patients with previous infections caused by the dengue virus, West Nile virus, yellow fever virus and Japanese encephalitis virus have been shown to show little or no detectable cross-reactivity to Zika virus NS1, in contrast to ELISAs based on the detection of antibodies to the Zika virus glycoprotein E [28]. The development of tests for detecting the NS1 protein of the dengue virus in serum has made it possible to easily and affordably diagnose patients with dengue fever in the acute phase [32]. However, there are currently no clinical tests for the detection of the NS1 protein of other flaviviruses, as it has previously been shown that NS1 secretion in flavivirus infection is low [33,34]. Nevertheless, the time interval at which the NS1 protein of West Nile virus can be detected in serum is similar to the time interval for the detection of the genomic RNA of this virus by RT-PCR.

The data obtained in this work also confirm the promising use of ELISA kits based on the NS1 proteins of flaviviruses from the TBEV complex for differential diagnosis, including the monitoring of viral encephalitis of unknown etiology. The development of more sensitive methods of NS1 protein detection, capable of detecting it even in low concentrations, may also allow for the use of such test systems for the diagnosis of flaviviruses, including those from the tick-borne encephalitis group. 

## 5. Conclusions

Currently, there is no reliable serological diagnosis to differentiate each virus of the *Flavivirus* family that is optimal, and the introduction of NS1-based serology can provide more accurate diagnosis and better epidemiologic support for the global spread of flaviviruses. In addition, the neutralization test is not suitable for OHFV diagnosis, as sera from TBEV-vaccinated humans and animals have previously been shown to obtain cross-reactive protective antibodies against OHFV [6,7].

In this work, the refolded recombinant OHFV NS1 protein was obtained for the first time, which retains antigenic properties similar to the native OHFV NS1 protein. It was indicated that IAFs from OHFV-infected mice did not recognize the TBEV NS1 protein, and mAbs to TBEV NS1 did not have cross-reactivity to OHFV NS1. In addition, it was shown for the first time that no cross-reactive antibodies to the NS1 protein of OHFV were detected in the sera of TBE patients containing antibodies to the NS1 protein of TBEV. Thus, this study indicated that the OHFV NS1 protein has an antigenic structure different from TBEV NS1 and could be potentially used as an antigen for the differential diagnosis of TBE and OHF. 

## Data Availability

Data are contained within the article and Supplementary Materials.

## Supplementary Materials

The following supporting information can be downloaded at https://www.mdpi.com/article/10.3390/v16071032/s1, Table S1. ELISA signals (OD_450_ nm) of sera from healthy donors; Table S2. ELISA signals (OD450 nm) of sera from patients with TBE.
