# Supplementary material for: Cross-Reactive Antibodies to the NS1 Protein of Omsk Hemorrhagic Fever Virus Are Absent in the Sera of Patients with Tick-Borne Encephalitis
Source: Viruses. 2024 Jun 27;16(7):1032. doi: 10.3390/v16071032 (PMC11281406; doi:10.3390/v16071032)
Supplement: Supplementary file 1 [file viruses-16-01032-s001.zip › viruses-3025339-supplementary.pdf]

Table S1. ELISA signals (OD_{450} nm) of sera from healthy donors.

Serum number	TBEV NS1, OD_{450} nm	OHFV NS1, OD_{450} nm
1	0,3	0,1
2	0,25	0,1
3	0,4	0,1
4	0,32	0,15
5	0,1	0,1
6	0,18	0,18
7	0,22	0,22
8	0,21	0,12
9	0,15	0,12
10	0,12	0,12
11	0,18	0,12
12	0,16	0,12
13	0,3	0,14
14	0,1	0,1
15	0,2	0,2
16	0,22	0,22
17	0,36	0,36
18	0,08	0,08
19	0,11	0,11
20	0,33	0,33
21	0,34	0,34
22	0,31	0,31
23	0,24	0,24
24	0,13	0,13
25	0,1	0,1
26	0,14	0,14
27	0,14	0,14
28	0,19	0,19
29	0,28	0,28
30	0,34	0,2
31	0,17	0,17
32	0,33	0,33
33	0,31	0,31
34	0,19	0,19
35	0,29	0,1
36	0,36	0,1
37	0,17	0,1
38	0,23	0,1
39	0,31	0,1
40	0,4	0,1
41	0,1	0,1
42	0,12	0,12
43	0,12	0,12
44	0,13	0,13

45	0,14	0,14
46	0,15	0,15
47	0,13	0,13
48	0,17	0,17
49	0,33	0,33
50	0,24	0,24
51	0,26	0,26
52	0,22	0,32
53	0,13	0,13
54	0,11	0,11
55	0,12	0,12
56	0,17	0,17
57	0,16	0,16
58	0,26	0,26
59	0,29	0,29
60	0,33	0,33
61	0,34	0,34
62	0,36	0,36
63	0,1	0,1
64	0,1	0,1
65	0,1	0,1
66	0,1	0,1
67	0,1	0,1
68	0,1	0,1
69	0,1	0,1
70	0,1	0,1
71	0,1	0,1
72	0,1	0,1
73	0,1	0,1
74	0,1	0,1
75	0,1	0,1
76	0,11	0,11
77	0,15	0,15
78	0,14	0,14
79	0,35	0,35
80	0,27	0,27
81	0,28	0,28
82	0,1	0,1
83	0,1	0,1
84	0,1	0,1
85	0,1	0,1
86	0,1	0,1
87	0,1	0,1
88	0,1	0,1
89	0,1	0,1
90	0,1	0,1

91	0,1	0,1
92	0,1	0,1
93	0,1	0,1
94	0,1	0,1
95	0,1	0,1
96	0,1	0,1

Table S2. ELISA signals (OD_{450} nm) of sera from patients with TBE

**Serum number**	**TBEV NS1, OD_{450} nm**	**OHFV NS1, OD_{450} nm**
1	2,6	0,3
2	1,1	0,22
3	2,8	0,1
4	0,4	
5	2,7	0,3
6	3	0,2
7	1,8	0,44
8	1,9	0,1
9	1,47	0,16
10	1	0,22
11	0,49	
12	1,9	0,23
13	2,6	0,18
14	2,3	0,11
15	3	0,12
16	2,1	0,19
17	1,4	0,24
18	1	0,23
19	0,9	0,19
20	0,85	0,17
21	2,6	0,16
22	2,4	0,8
23	2,2	0,25
24	2,9	0,23
